# Supplementary figures and images for: Recognition of galactose by a scaffold protein recruits a transcriptional activator for the GAL regulon induction in Candida albicans
Source: eLife. 2023 Feb 1;12:e84155. doi: 10.7554/eLife.84155 (PMC9925049; doi:10.7554/eLife.84155)

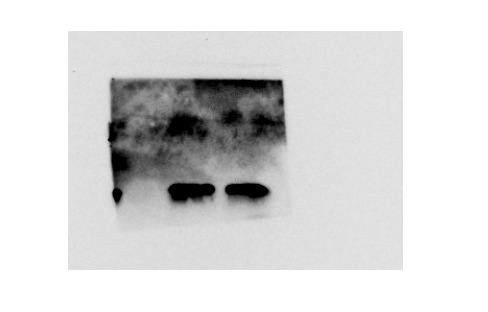

Supplement: Figure 1—figure supplement 3—source data 1. [file elife-84155-fig1-figsupp3-data1.zip › Figure 1- figure supplement 3- source data /unlabelled/TUBULIN.tif]

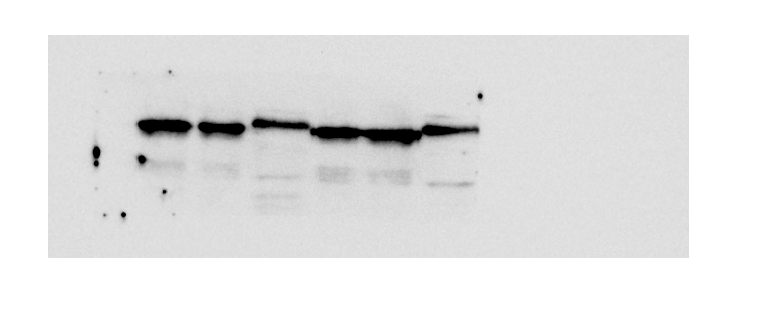

Supplement: Figure 1—figure supplement 3—source data 1. [file elife-84155-fig1-figsupp3-data1.zip › Figure 1- figure supplement 3- source data /unlabelled/FLAG.tif]

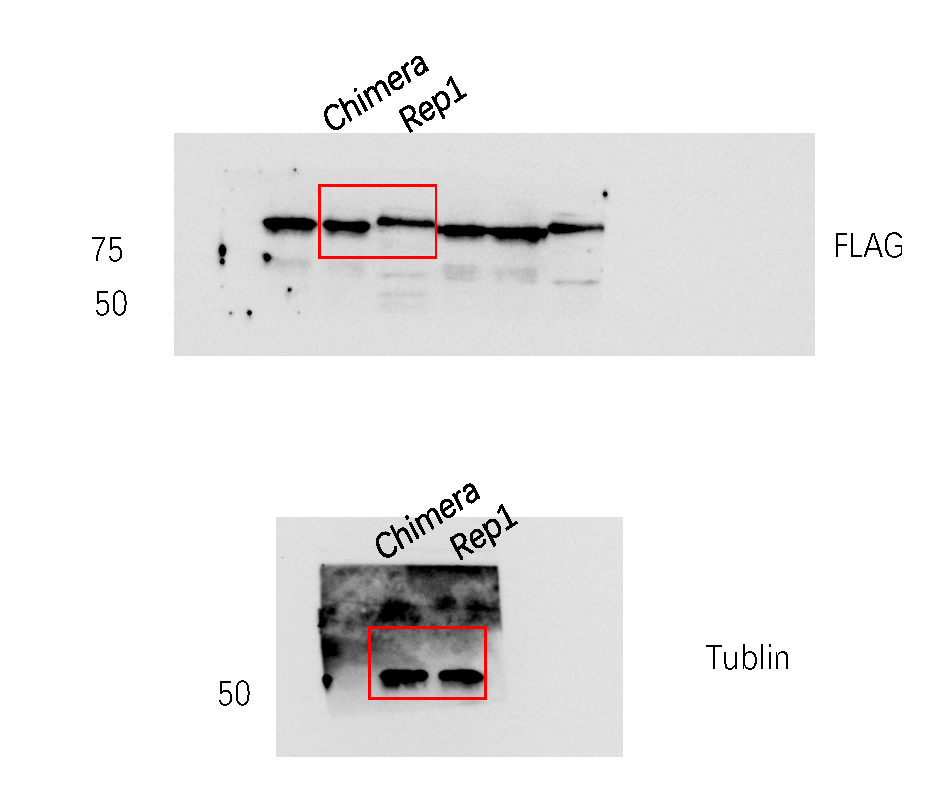

Supplement: Figure 1—figure supplement 3—source data 1. [file elife-84155-fig1-figsupp3-data1.zip › Figure 1- figure supplement 3- source data /labelled/LABELLED.tif]

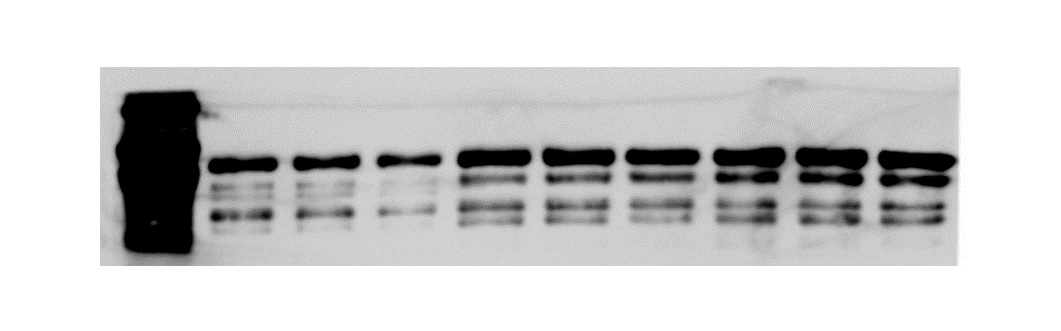

Supplement: Figure 2—source data 2. [file elife-84155-fig2-data2.zip › Figure 2- source data 2/unlabelled/input.tif]

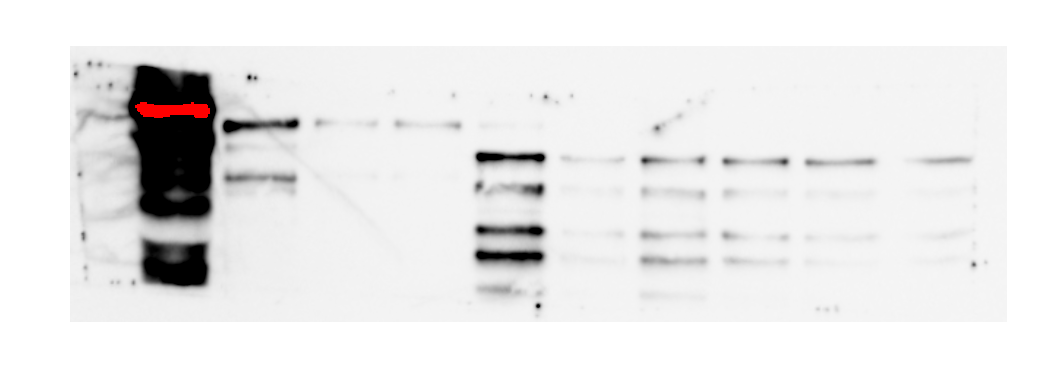

Supplement: Figure 2—source data 2. [file elife-84155-fig2-data2.zip › Figure 2- source data 2/unlabelled/bound.tif]

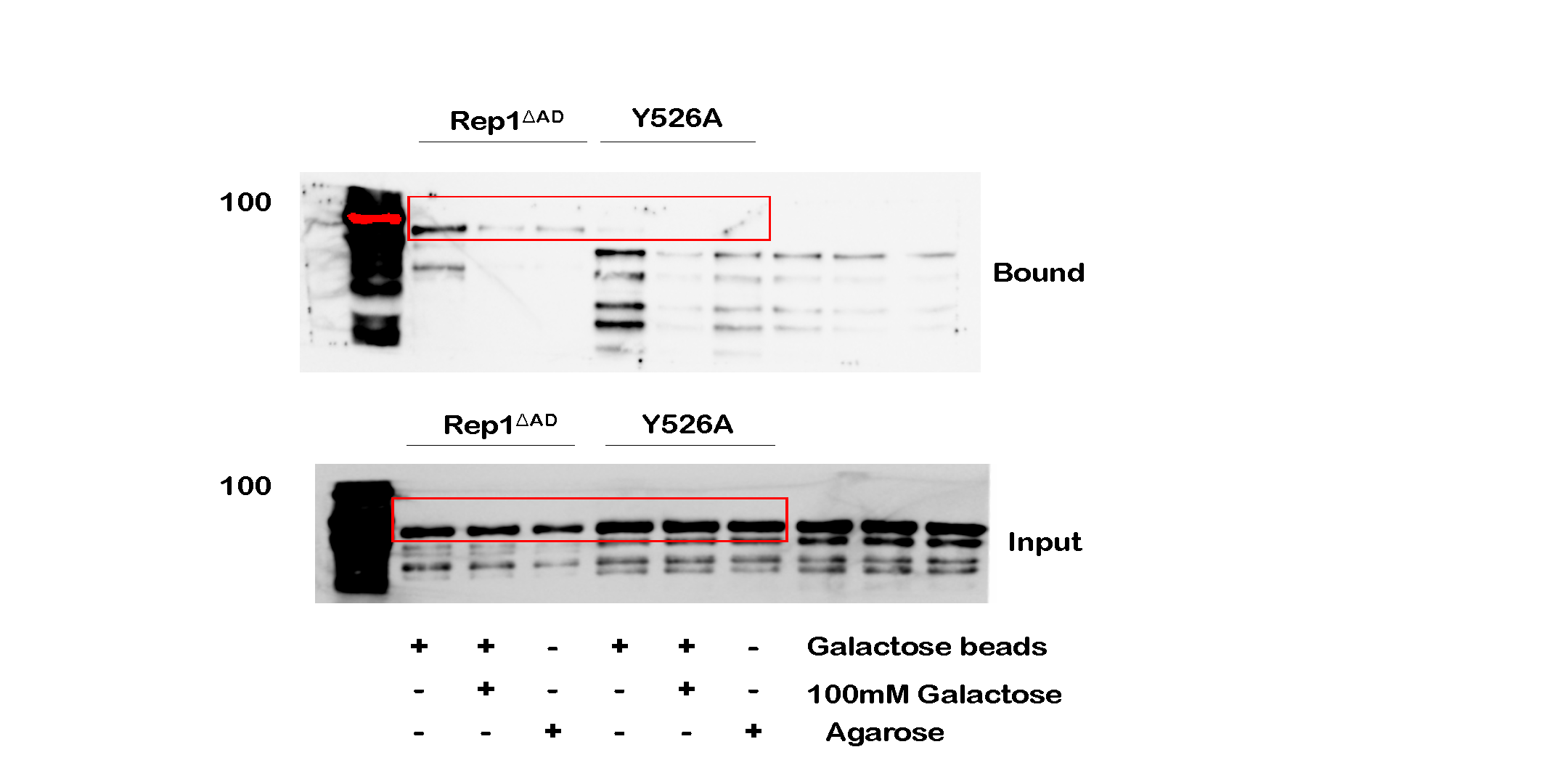

Supplement: Figure 2—source data 2. [file elife-84155-fig2-data2.zip › Figure 2- source data 2/labelled/labelled.tif]

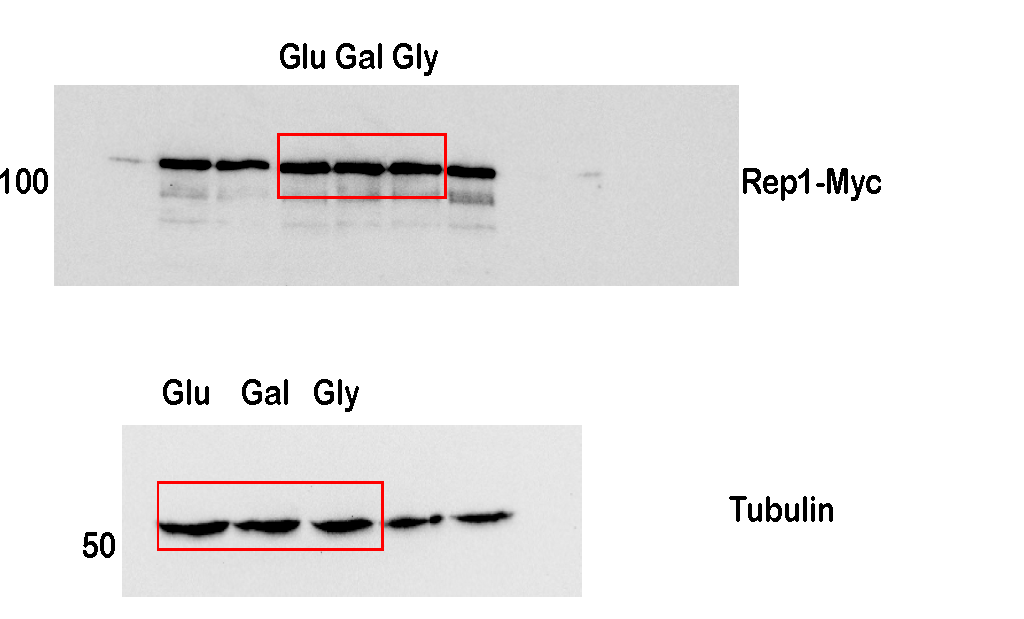

Supplement: Figure 2—figure supplement 1—source data 1. [file elife-84155-fig2-figsupp1-data1.zip › Figure 2- figure supplement 1- source data /labelled/A labelled.tif]

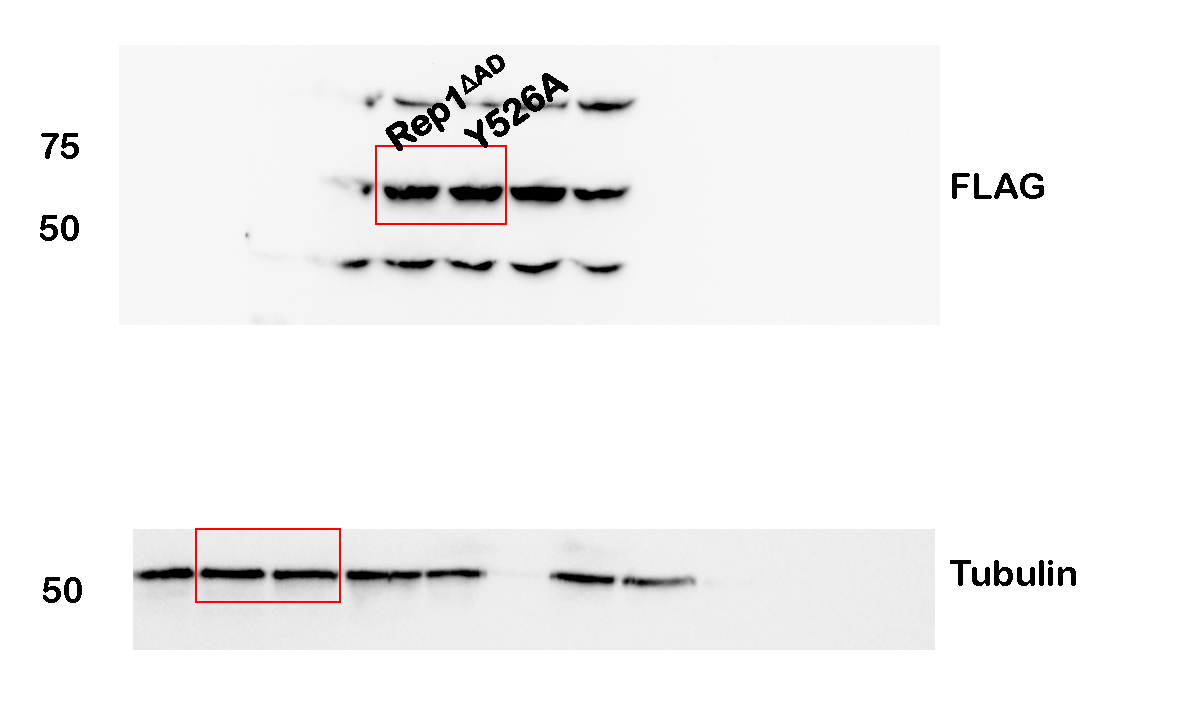

Supplement: Figure 2—figure supplement 1—source data 1. [file elife-84155-fig2-figsupp1-data1.zip › Figure 2- figure supplement 1- source data /labelled/C labelled.tif]

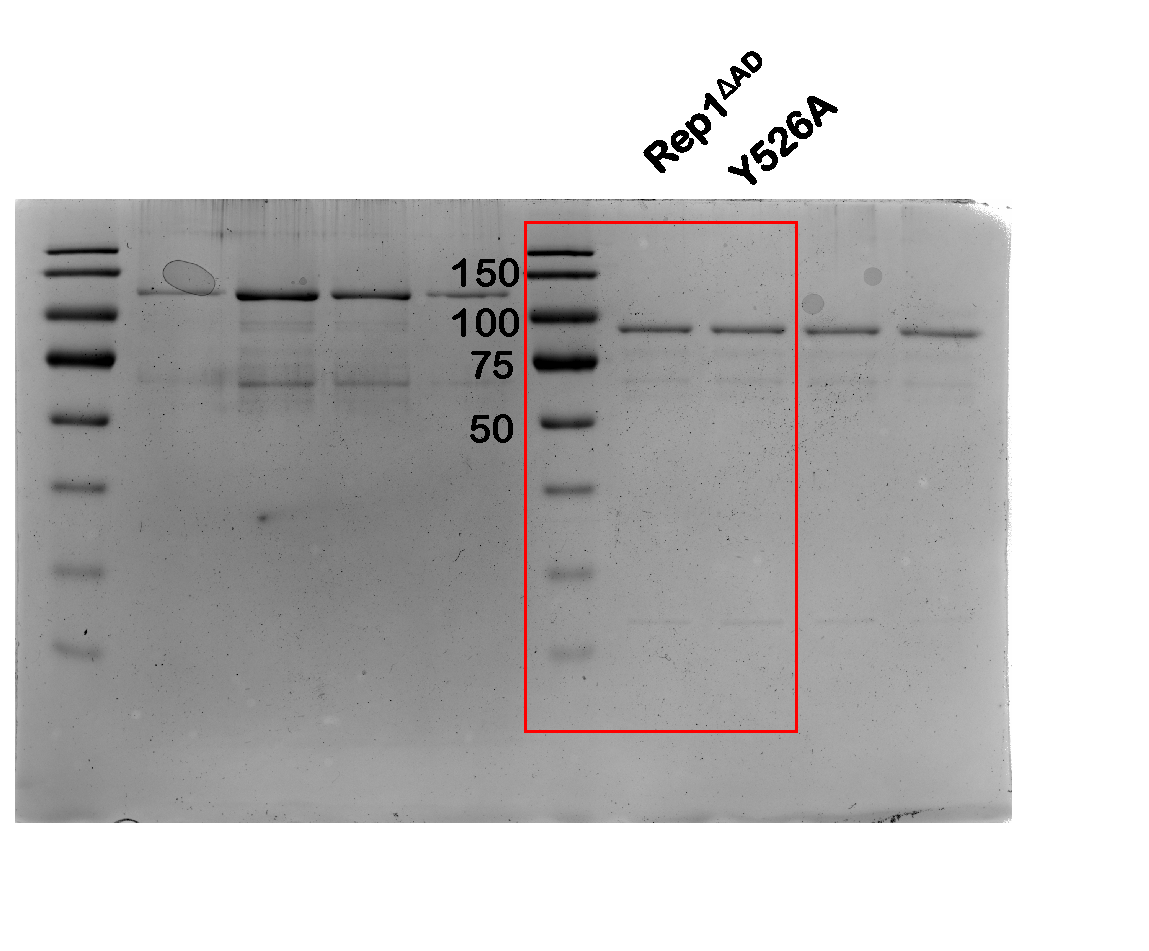

Supplement: Figure 2—figure supplement 1—source data 1. [file elife-84155-fig2-figsupp1-data1.zip › Figure 2- figure supplement 1- source data /labelled/D labelled.tif]

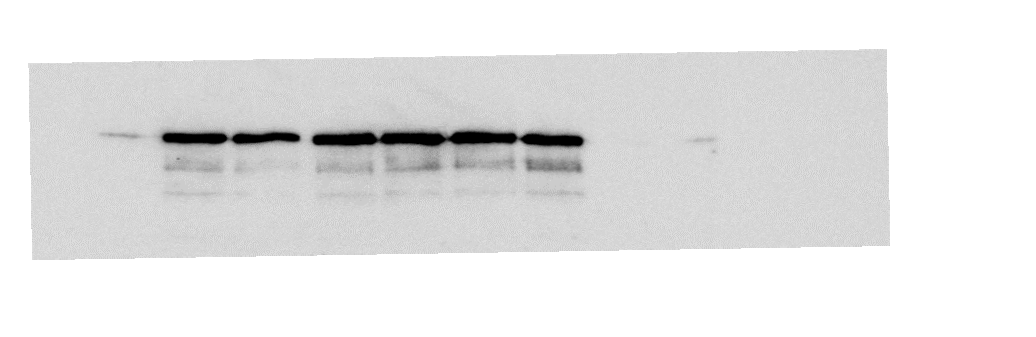

Supplement: Figure 2—figure supplement 1—source data 1. [file elife-84155-fig2-figsupp1-data1.zip › Figure 2- figure supplement 1- source data /unlabelled/A myc.tif]

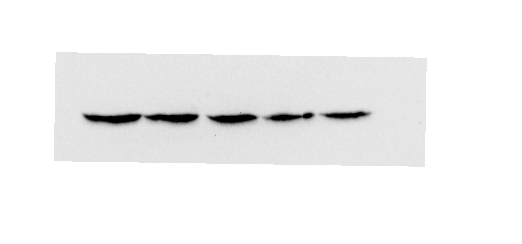

Supplement: Figure 2—figure supplement 1—source data 1. [file elife-84155-fig2-figsupp1-data1.zip › Figure 2- figure supplement 1- source data /unlabelled/A tubulin.tif]

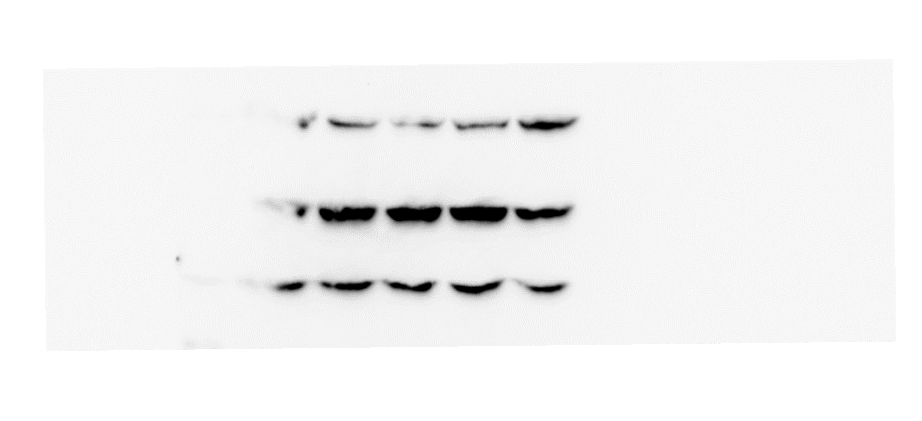

Supplement: Figure 2—figure supplement 1—source data 1. [file elife-84155-fig2-figsupp1-data1.zip › Figure 2- figure supplement 1- source data /unlabelled/C FLAG.tif]

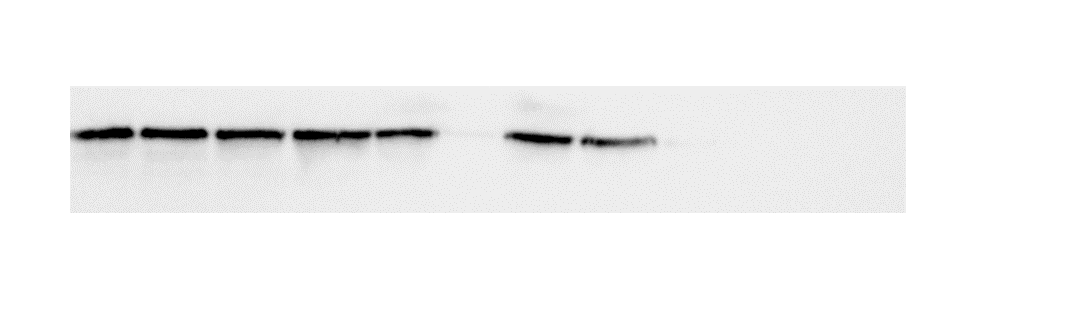

Supplement: Figure 2—figure supplement 1—source data 1. [file elife-84155-fig2-figsupp1-data1.zip › Figure 2- figure supplement 1- source data /unlabelled/C Tubulin.tif]

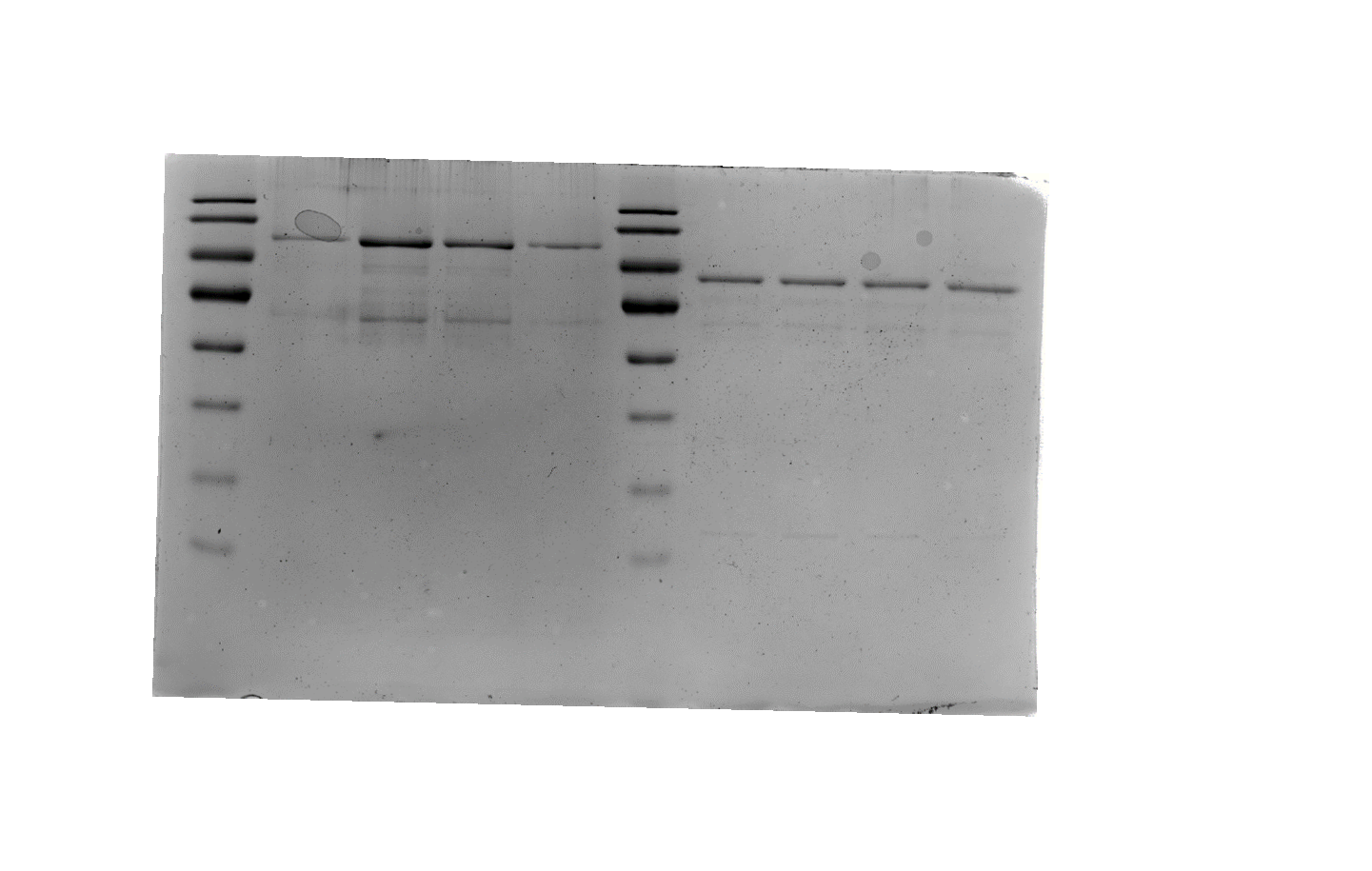

Supplement: Figure 2—figure supplement 1—source data 1. [file elife-84155-fig2-figsupp1-data1.zip › Figure 2- figure supplement 1- source data /unlabelled/D.tif]

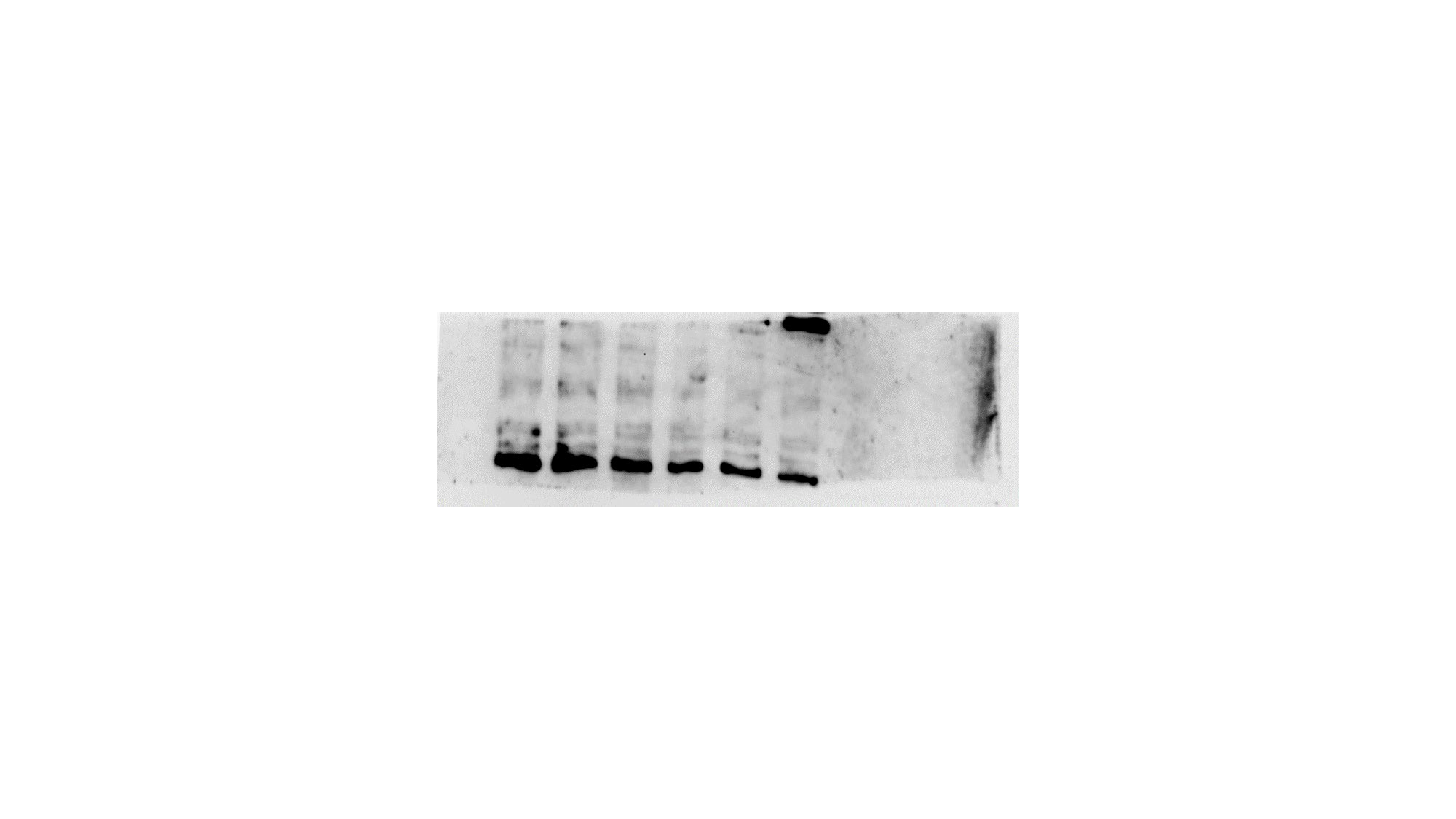

Supplement: Figure 3—source data 2. [file elife-84155-fig3-data2.zip › Figure 3- source data 2/unlabelled/3F INPUT.tif]

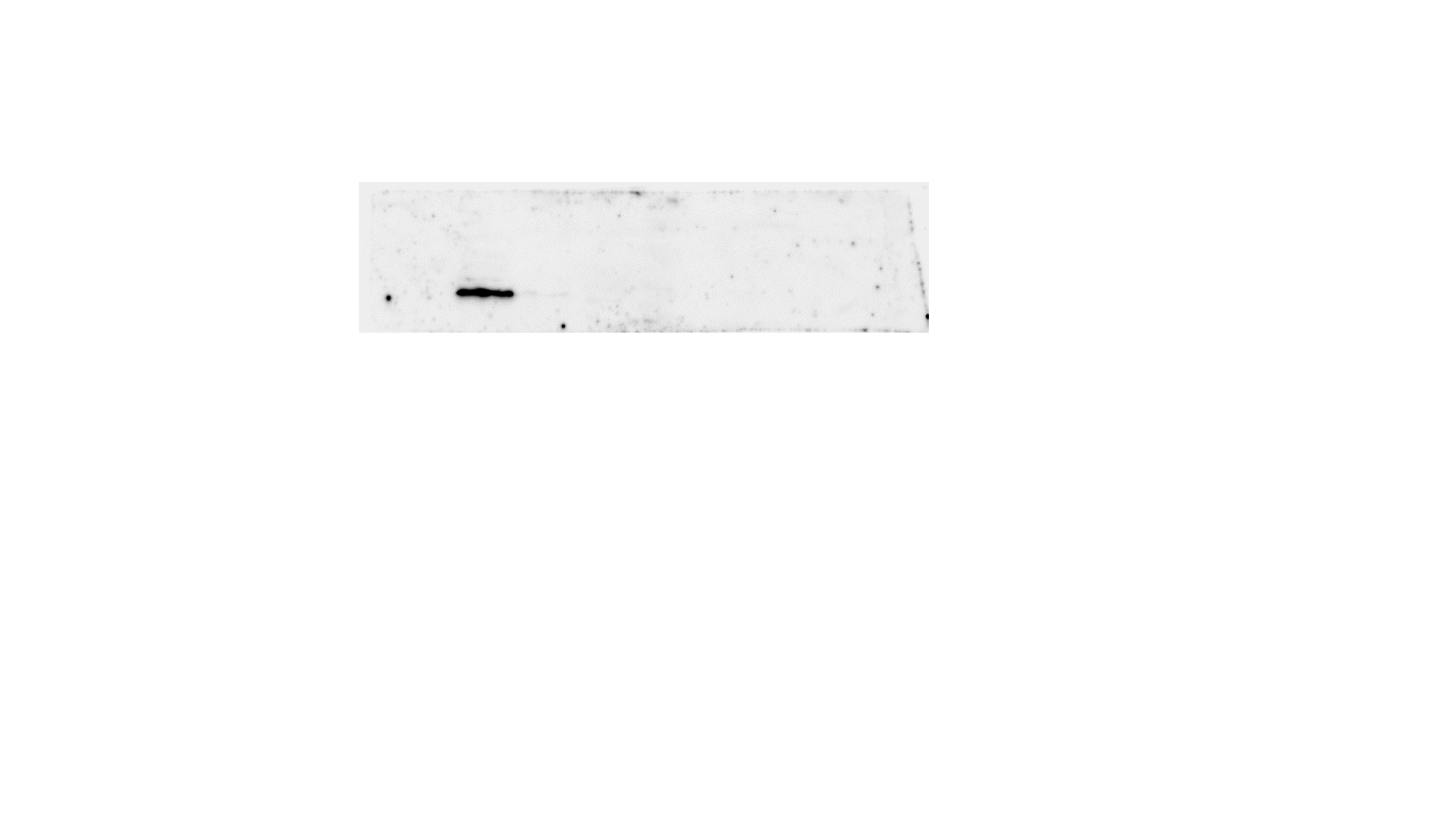

Supplement: Figure 3—source data 2. [file elife-84155-fig3-data2.zip › Figure 3- source data 2/unlabelled/3H IP.tif]

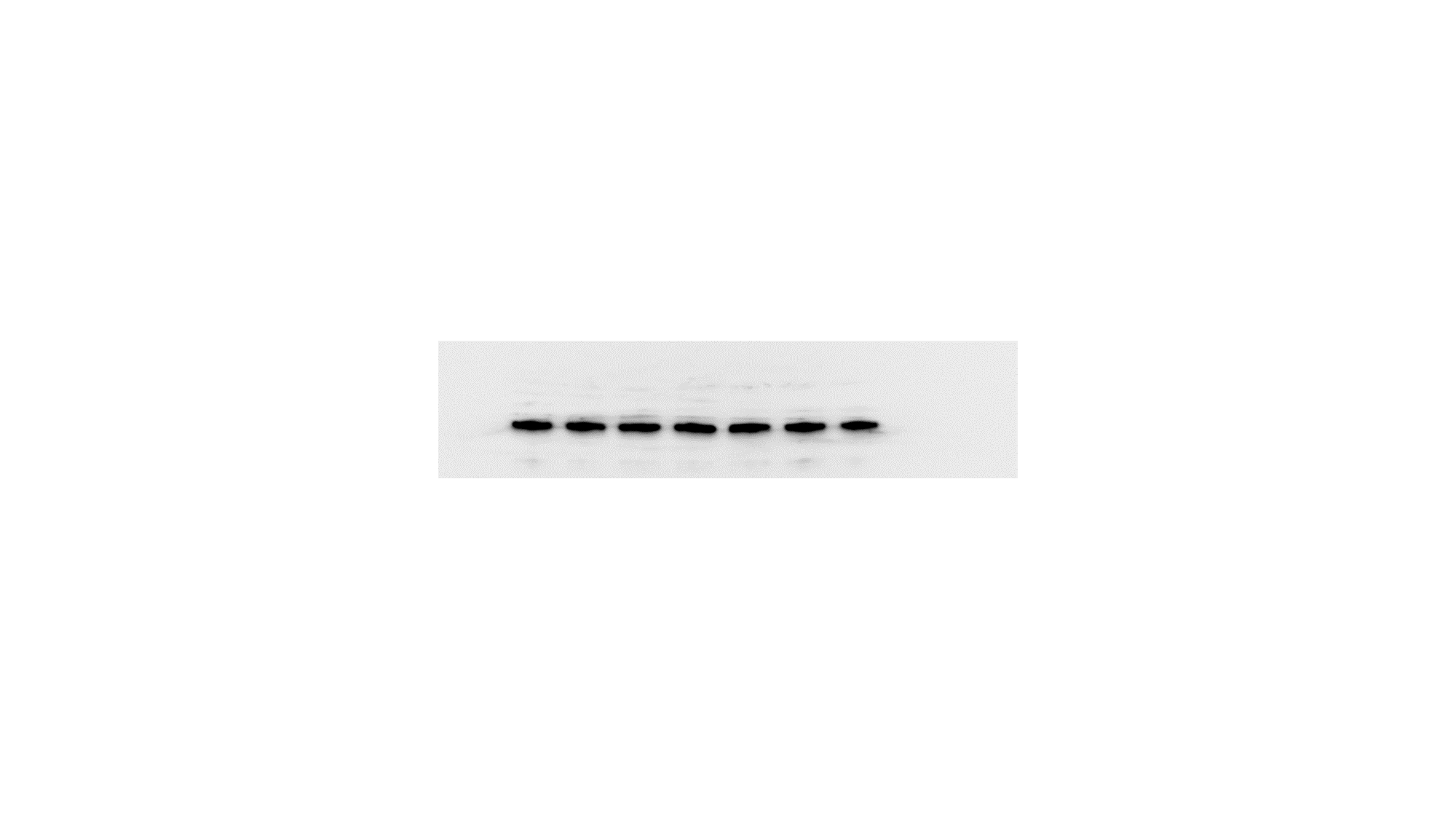

Supplement: Figure 3—source data 2. [file elife-84155-fig3-data2.zip › Figure 3- source data 2/unlabelled/3H input.tif]

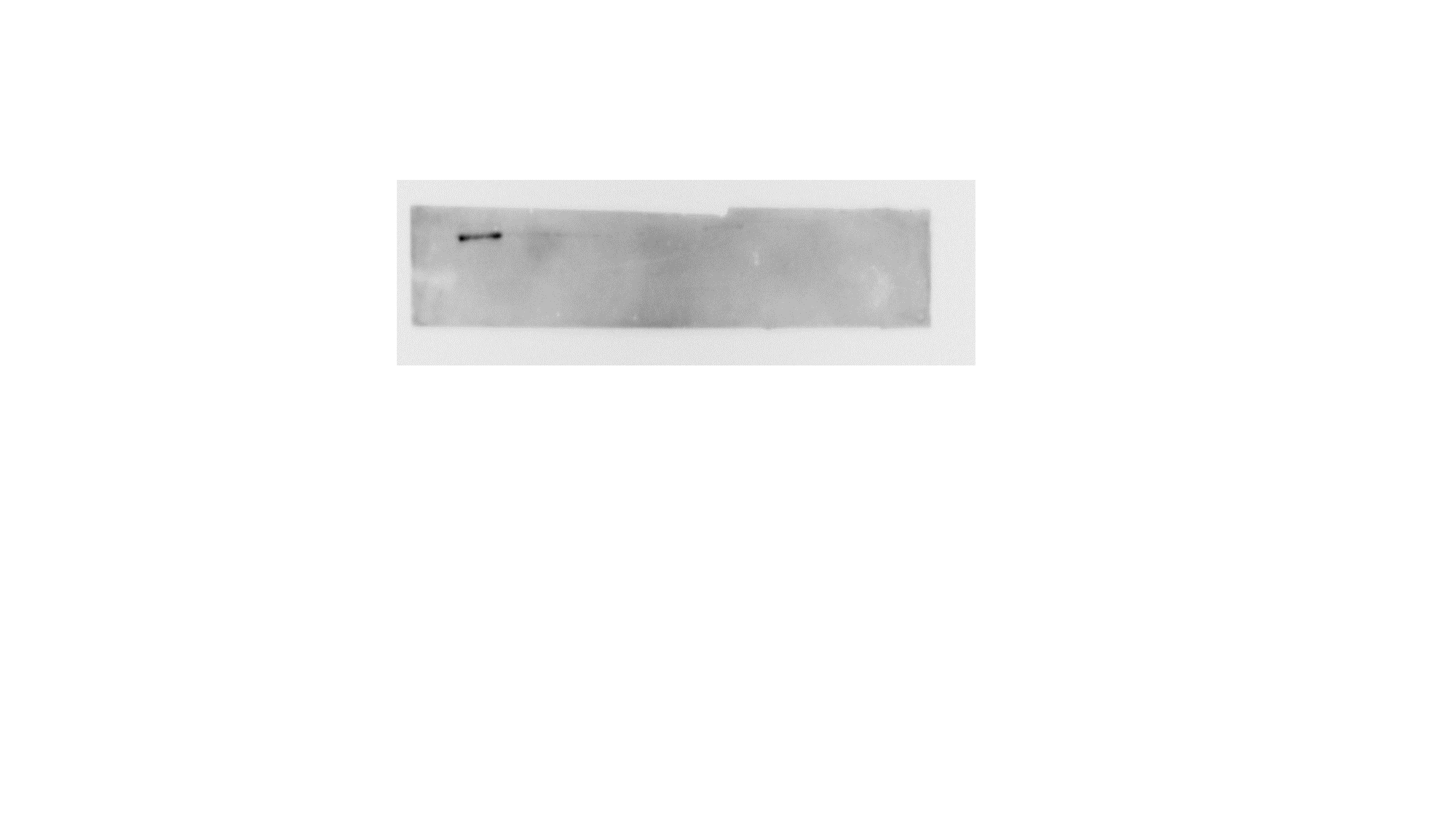

Supplement: Figure 3—source data 2. [file elife-84155-fig3-data2.zip › Figure 3- source data 2/unlabelled/3F IP.tif]

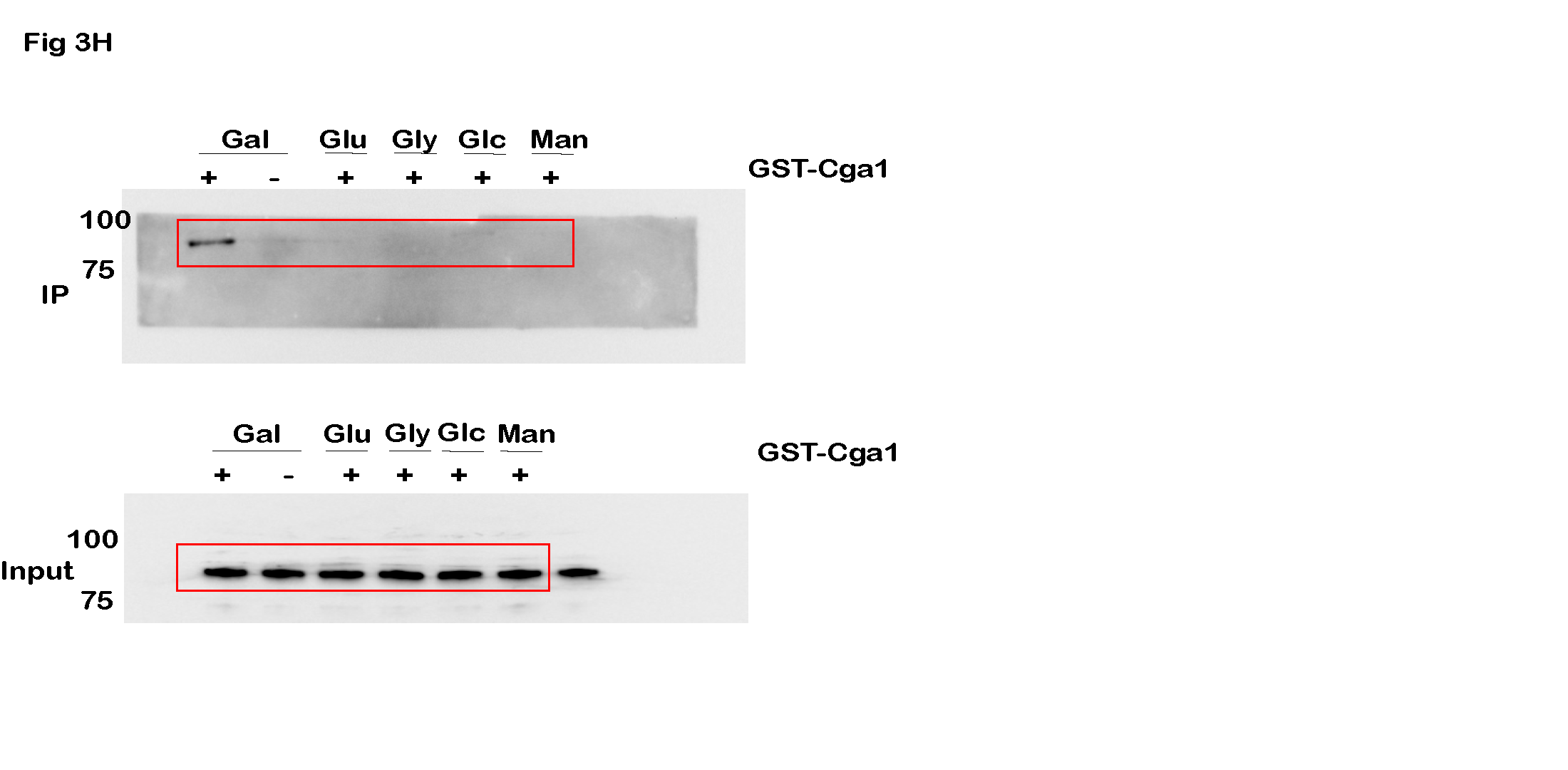

Supplement: Figure 3—source data 2. [file elife-84155-fig3-data2.zip › Figure 3- source data 2/labelled/3H labelled.tif]

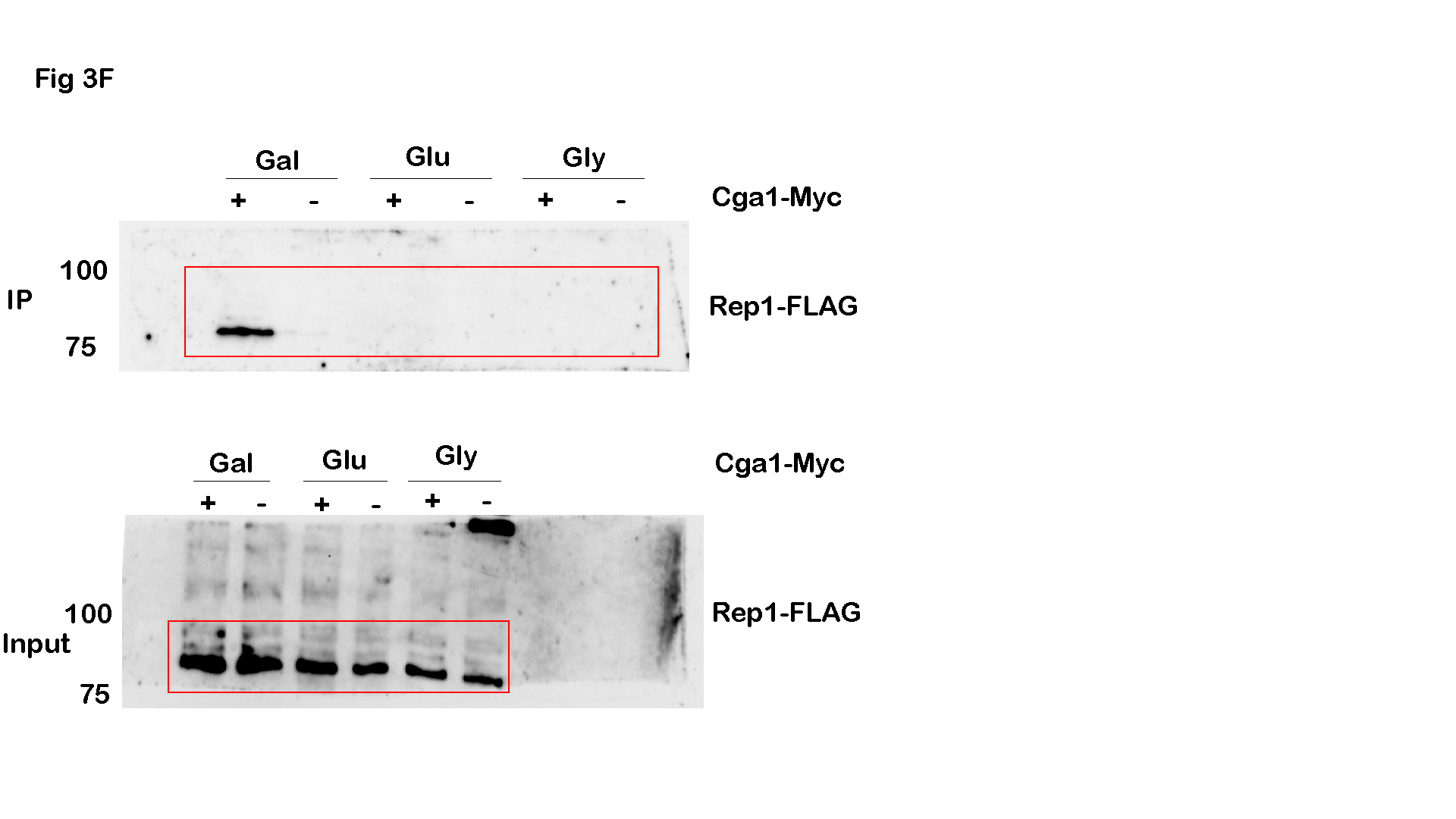

Supplement: Figure 3—source data 2. [file elife-84155-fig3-data2.zip › Figure 3- source data 2/labelled/3F LABELLED.tif]

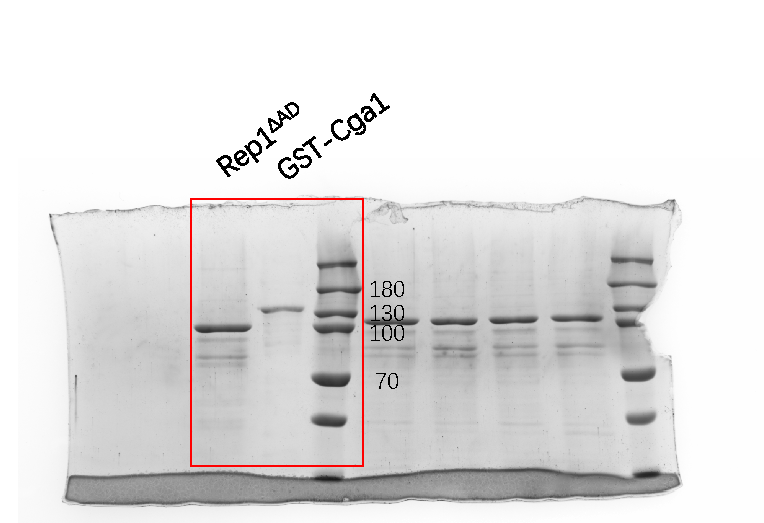

Supplement: Figure 3—figure supplement 1—source data 2. [file elife-84155-fig3-figsupp1-data2.zip › Figure 3- figure supplement 1- source data 2/labelled.tif]

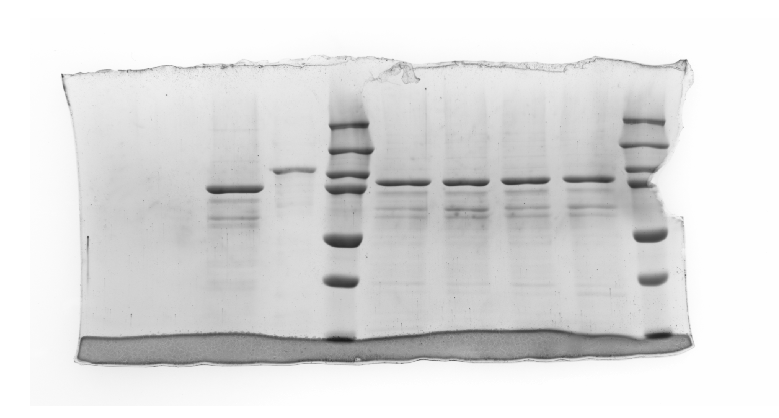

Supplement: Figure 3—figure supplement 1—source data 2. [file elife-84155-fig3-figsupp1-data2.zip › Figure 3- figure supplement 1- source data 2/unlabelled.tif]

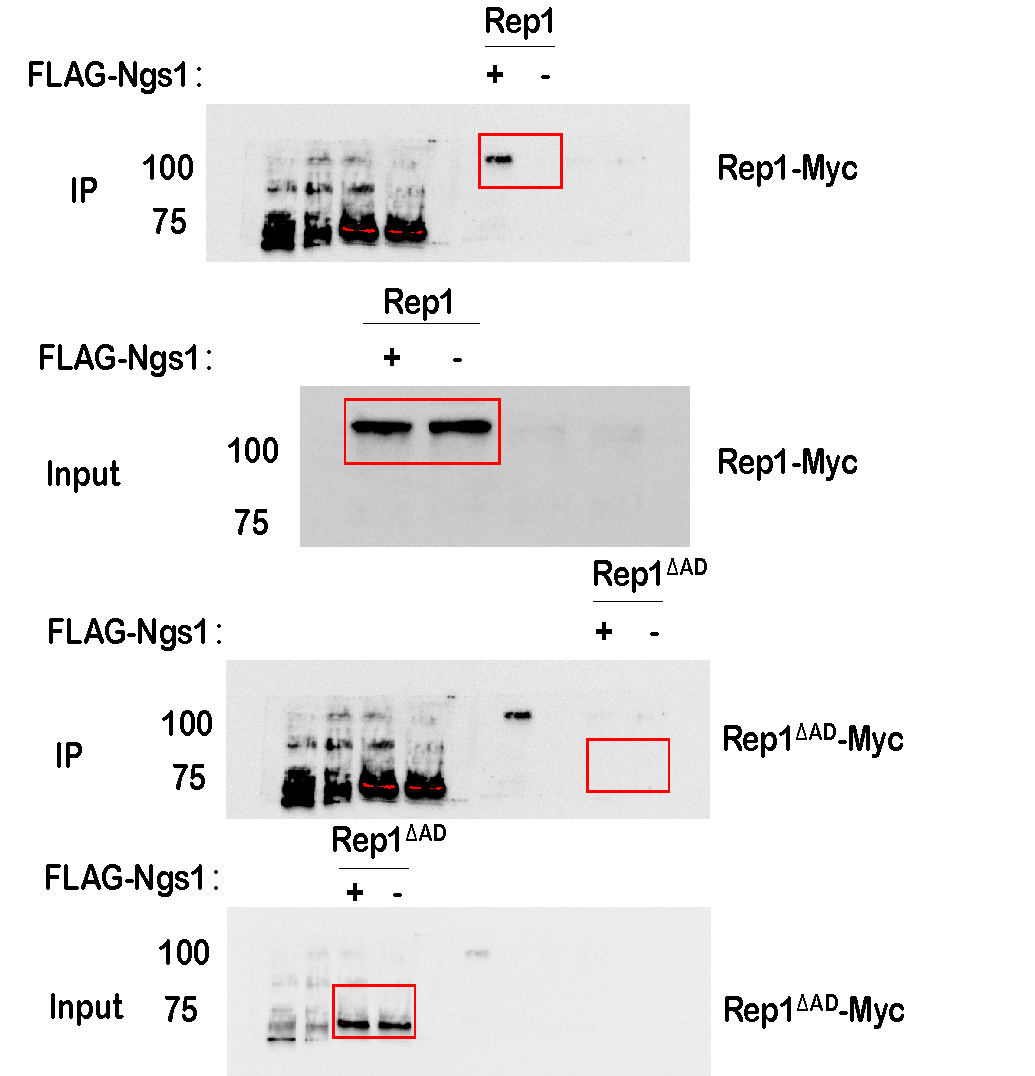

Supplement: Figure 4—source data 2. [file elife-84155-fig4-data2.zip › Figure 4- source data 2/labelled/labelled.tif]

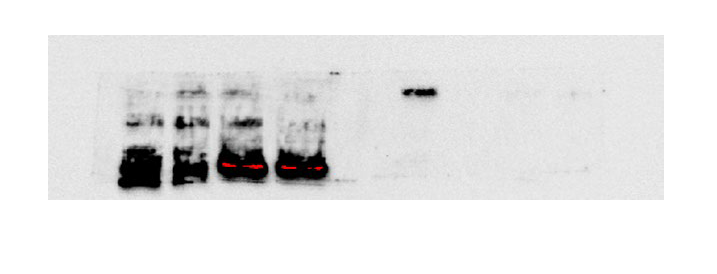

Supplement: Figure 4—source data 2. [file elife-84155-fig4-data2.zip › Figure 4- source data 2/unlabelled/ IP.tif]

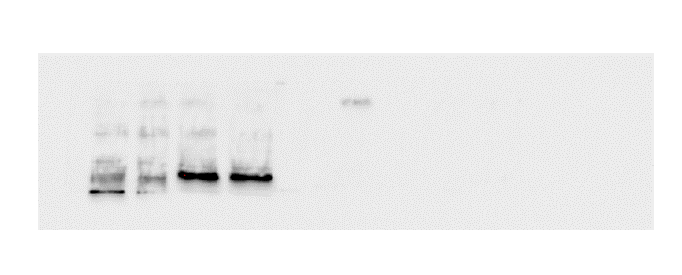

Supplement: Figure 4—source data 2. [file elife-84155-fig4-data2.zip › Figure 4- source data 2/unlabelled/Rep1 deltaAD input.tif]

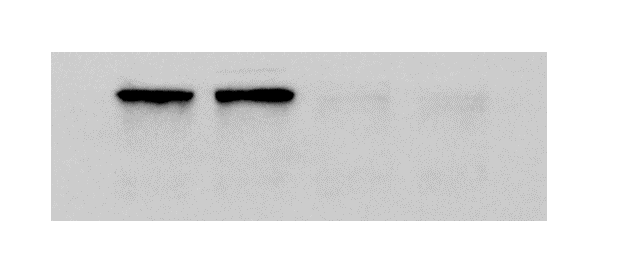

Supplement: Figure 4—source data 2. [file elife-84155-fig4-data2.zip › Figure 4- source data 2/unlabelled/Rep1 input.tif]
