# Supplementary material for: Recognition of galactose by a scaffold protein recruits a transcriptional activator for the GAL regulon induction in Candida albicans
Source: eLife. 2023 Feb 1;12:e84155. doi: 10.7554/eLife.84155 (PMC9925049; doi:10.7554/eLife.84155)
Supplement: Figure 3—source data 3. [file elife-84155-fig3-data3.zip › Figure 3 source data 3.pptx]

## Slide 1
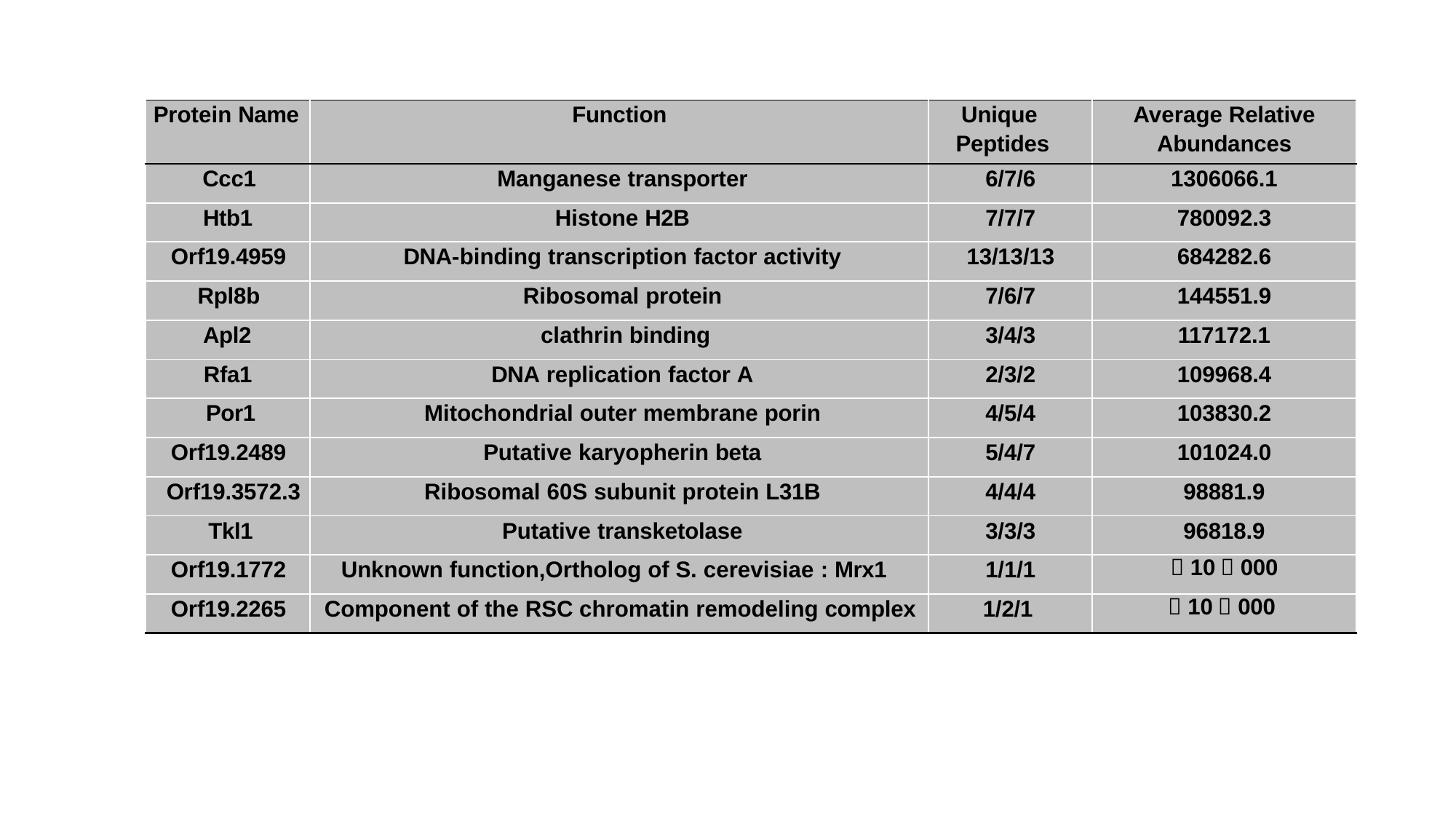

| Protein Name | Function | Unique Peptides | Average Relative Abundances |
| --- | --- | --- | --- |
| Ccc1 | Manganese transporter | 6/7/6 | 1306066.1 |
| Htb1 | Histone H2B | 7/7/7 | 780092.3 |
| Orf19.4959 | DNA-binding transcription factor activity | 13/13/13 | 684282.6 |
| Rpl8b | Ribosomal protein | 7/6/7 | 144551.9 |
| Apl2 | clathrin binding | 3/4/3 | 117172.1 |
| Rfa1 | DNA replication factor A | 2/3/2 | 109968.4 |
| Por1 | Mitochondrial outer membrane porin | 4/5/4 | 103830.2 |
| Orf19.2489 | Putative karyopherin beta | 5/4/7 | 101024.0 |
| Orf19.3572.3 | Ribosomal 60S subunit protein L31B | 4/4/4 | 98881.9 |
| Tkl1 | Putative transketolase | 3/3/3 | 96818.9 |
| Orf19.1772 | Unknown function,Ortholog of S. cerevisiae : Mrx1 | 1/1/1 | ＜10，000 |
| Orf19.2265 | Component of the RSC chromatin remodeling complex | 1/2/1 | ＜10，000 |
