# Supplementary material for: Recognition of galactose by a scaffold protein recruits a transcriptional activator for the GAL regulon induction in Candida albicans
Source: eLife. 2023 Feb 1;12:e84155. doi: 10.7554/eLife.84155 (PMC9925049; doi:10.7554/eLife.84155)
Supplement: Figure 4—source data 3. [file elife-84155-fig4-data3.zip › Figure 4 source data 3.pptx]

## Slide 1
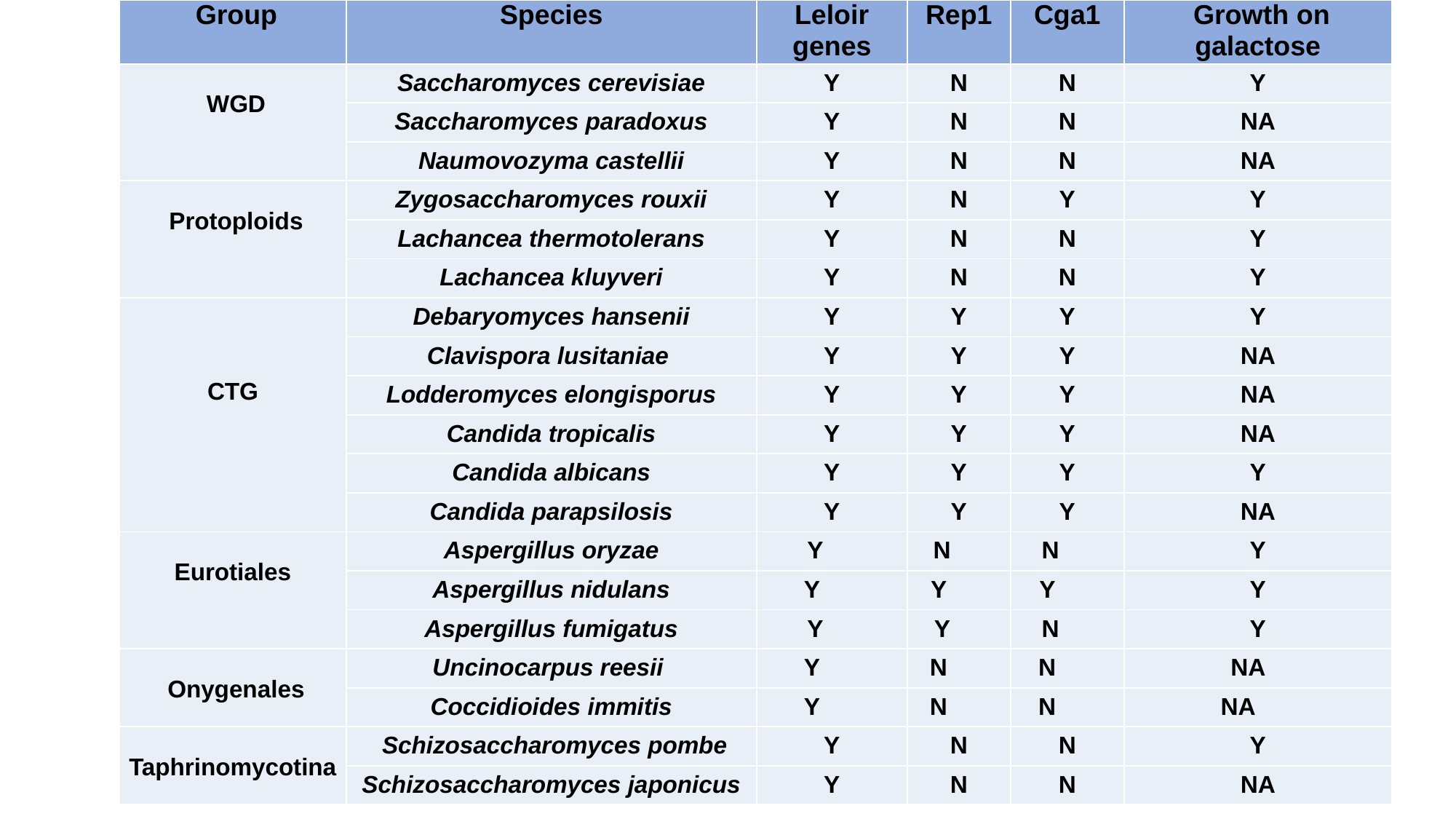

| Group | Species | Leloir genes | Rep1 | Cga1 | Growth on galactose |
| --- | --- | --- | --- | --- | --- |
| WGD | Saccharomyces cerevisiae | Y | N | N | Y |
| | Saccharomyces paradoxus | Y | N | N | NA |
| | Naumovozyma castellii | Y | N | N | NA |
| Protoploids | Zygosaccharomyces rouxii | Y | N | Y | Y |
| | Lachancea thermotolerans | Y | N | N | Y |
| | Lachancea kluyveri | Y | N | N | Y |
| CTG | Debaryomyces hansenii | Y | Y | Y | Y |
| | Clavispora lusitaniae | Y | Y | Y | NA |
| | Lodderomyces elongisporus | Y | Y | Y | NA |
| | Candida tropicalis | Y | Y | Y | NA |
| | Candida albicans | Y | Y | Y | Y |
| | Candida parapsilosis | Y | Y | Y | NA |
| Eurotiales | Aspergillus oryzae | Y | N | N | Y |
| | Aspergillus nidulans | Y | Y | Y | Y |
| | Aspergillus fumigatus | Y | Y | N | Y |
| Onygenales | Uncinocarpus reesii | Y | N | N | NA |
| | Coccidioides immitis | Y | N | N | NA |
| Taphrinomycotina | Schizosaccharomyces pombe | Y | N | N | Y |
| | Schizosaccharomyces japonicus | Y | N | N | NA |
