# Supplementary material for: Recognition of galactose by a scaffold protein recruits a transcriptional activator for the GAL regulon induction in Candida albicans
Source: eLife. 2023 Feb 1;12:e84155. doi: 10.7554/eLife.84155 (PMC9925049; doi:10.7554/eLife.84155)
Supplement: Supplementary file 1. [file elife-84155-supp1.docx]

**Supplementary File 1A**

***Candida albicans* strains used in this study.**

| **Strains** | **Genotype** | **Sources** |
| --- | --- | --- |
| BWP17 | *ura3Δ::imm434/ura3Δ::imm434*, *his1Δ::hisG/his1Δ::hisG, arg4Δ::hisG/arg4Δ::hisG* | (45) |
| SC5314 | Wild type | (48) |
| CAI4 | *ura3Δ::imm434/ura3Δ::imm434* | (48) |
| YLO141 | *ura3Δ::imm434/ura3Δ::imm434, his1Δ::hisG/his1Δ::hisG, arg4Δ::hisG/arg4Δ::hisG rep1Δ::ARG4/rep1Δ::HIS1* | (25) |
| *ngs1* | *his1Δ/his1Δ,leu2Δ/leu2Δ,arg4Δ/arg4Δ,URA3/ura3Δ::imm434, IRO1/iro1Δ::imm434, ngs1Δ::C.d. HIS1/ngs1Δ::C.m. LEU2* | (26) |
| *cga1* | *ura3Δ::imm434/ura3Δ::imm434, his1Δ::hisG/his1Δ::hisG,*  *arg4Δ::hisG/arg4Δ::hisG,* *cga1Δ::ARG4/cga1Δ::URA3/ cga1Δ::HIS1* | This study |
| *orf19.2265* | *his1Δ/his1Δ,leu2Δ/leu2Δ,arg4Δ/arg4Δ,URA3/ura3Δ::imm434, IRO1/iro1Δ::imm434,orf19.2265Δ::C.d.HIS1/orf19.2265Δ::C.m. LEU2* | (26) |
| *orf19.1772* | *leu2*Δ*::C.m.LEU2/leu2*Δ*::C.d.HIS1, ura3*Δ*/URA3, his1*Δ*/his1*Δ*, arg4*Δ*/ arg4*Δ*::ARG4, iro1*Δ*/IRO1, orf19.1772*Δ*/orf19.1772*Δ*, Neut5L::FRT/Neut5L* | This study |
| Ca672 | *ura3Δ::imm434/ura3Δ::imm434*, *ade2::hisG/ade2::hisG::*  [pOPlacZ] | (31) |

**Supplementary File 1B**

**Plasmids used in this study.**

| **Description** | **Purpose** | **Sources** |
| --- | --- | --- |
| pBA1 |  | (49) |
| pBA1-REP1 |  | This study |
| pBA1-REP1 (AD) |  |  |
| pBA1-REP1 (BD) | ADH1 promoter for |  |
| pBA1-REP1 (C) | complementation assays  in *rep1* mutant |  |
| pBA1-REP1^ΔAD^ |  |  |
| pBA1-REP1^ΔC^ |  |  |
| pBA1-Chimera |  |  |
| pBA1-REP1^ΔAD Y526A^ |  |  |
| pET28a |  | From novagen |
| pET28a-REP1^ΔAD^ | Protein purification for pull down and ITC | This study |
| pET28a- REP1^ΔAD Y526A^ |  |  |
| ACT1p-REP1-Myc | ChIP and ChIP-Seq of Rep1 | This study |
| ACT1p-CGA1-Myc | ChIP of Cga1 | This study |
| pRHO1-GFP |  | (50) |
| pRHO1-GFP-REP1 | Rep1 localization and GFP purification for mass spectrometry | This study |
| pRHO1-GFP-CGA1 | Cga1 localization | This study |
| ACT1p-REP1-Myc | In vivo CO-IP assay | This study |
| ADH1p-3FLAG-NGS1 |  | (8) |
| ACT1p- REP1^ΔAD^ -Myc |  | This study |
| pGEX-GST-CGA1 | In vitro CO-IP assay | This study |
| Clp-lexA |  | (31) |
| Clp-lexA-REP1 | β-galactosidase assays | This study |

**Supplementary File 1C**

**Primers used in this study.**

| Primer | Sequence (5’-3’) | Purpose and features | |
| --- | --- | --- | --- |
| 1 | CAACAACAAATACAAAAACAAAGATCTGCCACACCCACATGGATTC | pBA1-REP1 (AD) | ADH1 promoter for  complementation assays  in *rep1* mutant |
| 2 | ATACGACTCACATAGGGCGAATTGGGTACCTCAGCCACTTTGGGTATCCATAC |  |  |
| 3 | CAACAACAAATACAAAAACAAAGATCTATGTCATCGACTCCAAAGAGG | pBA1-REP1 (BD) |  |
| 4 | ATACGACTCACTATAGGGCGAATTGGGTACCTCAGTTTCTTTAGCGTAGAAGC |  |  |
| 5 | CAACAACAAATACAAAAACAAAGATCTATGGATATTCTAATCAAGGGTCG | pBA1-REP1(C) |  |
| 6 | ATACGACTCACTATAGGGCGAATTGGGTACCGTCTCGGCATTGTCTTTTTC |  |  |
| 7 | CAACAACAAATACAAAAACAAAGATCTGCCACACCCACATGGATTC | pBA1-REP1^ΔC^ |  |
| 8 | ATACGACTCACTATAGGGCGAATTGGGTACCTCAGTTTCTTTAGCGTAGAAGC |  |  |
| 9 | CAACAACAAATACAAAAACAAAGATCTATGTCATCGACTCCAAAGAGG | pBA1-REP1^ΔAD^ |  |
| 10 | ATACGACTCACTATAGGGCGAATTGGGTACCGTCTCGGCATTGTCTTTTTC |  |  |
| 11 | CAACAACAAATACAAAAACAAAGATCTGCCACACCCACATGGATTC | pBA1-REP1 |  |
| 12 | ATACGACTCACTATAGGGCGAATTGGGTACCGTCTCGGCATTGTCTTTTTC |  |  |
| 13 | CAACAACAAATACAAAAACAAAGATCTATGCAAGCTGACGCTCAATCACAAG | pBA1-Chimera |  |
| 14 | GATTAGAATATCGTTTCTTTCAGCGTAGCTTGACGGAGATCTGCC |  |  |
| 15 | AGAGGCAGATCTCCGTCAAGCTACGCTGAAAGAAACGATATTC |  |  |
| 16 | GTCTTTGTAGTCGATATCTGTACGCGTTTTGAAATAAACATTGGAAC |  |  |
| 17 | CAACAACAAATACAAAAACAAAGATCTATGGATTCAAATAAAGAGAGA | pBA1-REP1^ΔAD Y526A^ |  |
| 18 | GTCTTTGTAGTCGATATCTGTACGCGTTTTGAAATAAACATTGGAACT |  |  |
| 19 | AACCCAAGCTTCGCCGCTGAAAGAAACGATATT |  |  |
| 20 | GTTTCTTTCAGCGGCGAAGCTTGGGTTACGACC |  |  |
| 23 | CAACAACAAATACAAAAACAAAGATCTATGGATTCATCCAAAGATAG | pBA1-*Ct*REP1 |  |
| 24 | GTCTTTGTAGTCGATATCTGTACGCGTTTACTTGAAATAAACGTTTG |  |  |
| 25 | CAACAACAAATACAAAAACAAAGATCTATGTCGAATCAAGAAGAAGA | pBA1-*Ct*CGA1 |  |
| 26 | CGACGGTATCGATAAGCTTGATATCTTATTCACCAACTAATCTGC |  |  |
| 27 | CAACAACAAATACAAAAACAAAGATCTATGAACTCCAGTGATAGGAC | pBA1-*Cp*REP1 |  |
| 28 | CGACGGTATCGATAAGCTTGATATCCTATTTAAAATACACATTTG |  |  |
| 29 | CAACAACAAATACAAAAACAAAGATCTATGTCTGCTGCTGAAGATGA | pBA1-*Cp*CGA1 |  |
| 30 | CGACGGTATCGATAAGCTTGATATCTTATTCTCCAACCAAACGAG |  |  |
| 21 | TAAAAATCGGTTTGTATGTG | *GAL1/GAL10* promotor | qRT-PCR analysis |
| 22 | CACAATGTTTAGACATCTGA |  |  |
| 23 | GTAGTGTGTGCACTGCC | *ACT1* promoter |  |
| 24 | CCACCGTCCATTTTGAATGA |  |  |
| 25 | TGGATTAGCTCGAGCATTTG | *CDC28*-qRT-PCR |  |
| 26 | CCAACAGACCACATATCTACCC |  |  |
| 27 | TCACTGCTGAAGGTAATTTG | *CGA1*-qRT-PCR |  |
| 28 | TTTATGACGATAACGTCCAC |  |  |
| 29 | GGTCCACGCGTGGTGGAGGTCCAGGTGGAGATTCAAATAAAGAGAGAAT | Clp-lexA-Rep1 | β-galactosidase assays |
| 30 | ACATGCATGCTCATTTGAAATAAACATTGG |  |  |
| 31 | GGTCCACGCGTGGTGGAGGTCCAGGTGGACATCCATCAGCTGGTGTCAA | Clp-lexA-Ndt80 |  |
| 32 | TCCCCCGGGTTACTGTGGAGGAGTAGGGG |  |  |
| 33 | GGTCCACGCGTGGTGGAGGTCCAGGTGGATCTAATCACGAAGATGATTA | Clp-lexA-Cga1 |  |
| 34 | ACATGCATGCTTACTCACCAACTAATCTAC |  |  |
| 35 | CTCACAGAGAACAGATTGGTGGATCCATGAGCCGCAAACGCGATGTGAA | pET28a-REP1^ΔAD^ | Protein purification for |
| 36 | TCAGTGGTGGTGGTGGTGGTGCTCGAGTTATTTAAAATACACGTTGC |  |  |
| 37 | AACCCAAGCTTCGCCGCTGAAAGAAACGATATT | pET28a- REP1^ΔAD Y526A^ | pull down and ITC |
| 38 | GTTTCTTTCAGCGGCGAAGCTTGGGTTACGACC |  |  |
| 39 | GGTATGGATGAATTGTACAAACTGCAGATGGATTCAAATAAAGAGAG | pRHO1-GFP-REP1 | Rep1 localization and GFP purification for mass spectrometry |
| 40 | AAGCGCTTTCAGTTTCTCCATGGATCCTCATTTGAAATAAACATTGG |  |  |
| 41 | CATAAGCTTTATTAAAATGTCTAAAGGTG | pRHO1-GFP | GFP purification for mass spectrometry |
| 42 | CGGGATCCTTATTTGTACAATTCATCCAT |  |  |
| 43 | GGTATGGATGAATTGTACAAACTGCAGATGTCTAATCACGAAGATGA | pRHO1-GFP-CGA1 | Cga1 localization |
| 44 | AAGCGCTTTCAGTTTCTCCATGGATCCTTACTCACCAACTAATCTAC |  |  |
| 45 | TTAAAAAAATATAATCATGCAAAGGATCCATGTCTAATCACGAAGATGA | ACT1p-CGA1-Myc | ChIP of Cga1 |
| 46 | GTTCACCGTTAATTAACCCGGGAACGCGTGCCTCACCAACTAATCTACTAT |  |  |
| 47 | TTAAAAAAATATAATCATGCAAAGGATCCATGTCATCGACTCCAAAGAGG | ACT1p-REP1-Myc | ChIP and ChIP-Seq of Rep1 |
| 48 | GTTCACCGTTAATTAACCCGGGAACGCGTGCTTTGAAATAAACATTGGAACTC |  |  |
| 49 | TTAAAAAAATATAATCATGCAAAGGATCCATGGATTCAAATAAAGAGAG | ACT1p- REP1ΔAD -Myc | In vivo CO-IP assay |
| 50 | GTTCACCGTTAATTAACCCGGGAACGCGTGCTTTGAAATAAACATTGGAACTC |  |  |
| 51 | AAGTTCTGTTCCAGGGGCCCCTGGGATCCATGTCTAATCACGAAGATGA | pGEX-GST-CGA1 | In vitro CO-IP assay |
| 52 | GTCAGTCAGTCACGATGCGGCCGCTCGAGTTACTCACCAACTAATCTAC |  |  |
| 53 | CCACTGGTAGAAGACAAAAGACTCGTAGAGGGATTTCTACTAATGATTCATCGGTAGTTGTGTGGAATTGTGAGCGGATA |  | CGA1 disruption |
| 54 | TGTTGATATTGAGATTCGTCTAGTTGTTGTTGAGCAGCAGCAGCAGCTTGAGCTTGAGTATTTCCCAGTCACGACGTT |  |  |

Restriction sites are underlined.
